# Classification of unsequenced Mycobacterium tuberculosis strains in a high-burden setting using a pairwise logistic regression approach

**DOI:** 10.1099/acmi.0.000964.v3

**Published:** 2025-05-12

**Authors:** Isabel Rancu, Benjamin Sobkowiak, Joshua L. Warren, Nelly Ciobanu, Alexandru Codreanu, Valeriu Crudu, Caroline Colijn, Ted Cohen, Melanie H. Chitwood

**Affiliations:** 1 Department of Epidemiology of Microbial Diseases, Yale School of Public Health, New Haven, Connecticut, USA; 2 Department of Biostatistics, Yale School of Public Health, New Haven, Connecticut, USA; 3 Phthisiopneumology Institute, Strada Constantin Vârnav 13, Chisinau, Republic of Moldova; 4 Department of Mathematics, Simon Fraser University, Burnaby, Canada

**Keywords:** classification, epidemiology, tuberculosis

## Abstract

Over the past three decades, molecular epidemiological studies have provided new opportunities to investigate the transmission dynamics of *Mycobacterium tuberculosis*. In most studies, a sizable fraction of individuals with notified tuberculosis cannot be included, either because they do not have culture-positive disease (and thus do not have specimens available for molecular typing) or because resources for conducting sequencing are limited. A recent study introduced a regression-based approach for inferring the membership of unsequenced tuberculosis cases in transmission clusters based on host demographic and epidemiological data. This method was able to identify the most likely cluster to which an unsequenced strain belonged with an accuracy of 35%, although this was in a low-burden setting where a large fraction of cases occurred among foreign-born migrants. Here, we apply a similar model to *M. tuberculosis* whole-genome sequencing data from the Republic of Moldova, a setting of relatively high local transmission. Using a maximum cluster span of ~40 single nucleotide polymorphisms (SNPs) and a cluster size cutoff of *n*≥10, we could best predict the specific cluster to which each clustered case was most likely to be a member with an accuracy of 17.2 %. In sensitivity analyses, we found that a more restrictive (~20 SNPs threshold) or permissive (~80 SNPs) threshold did not improve performance. We found that increasing the minimum cluster size improved prediction accuracy. These findings highlight the challenges of transmission inference in high-burden settings like Moldova.

**Impact Statement**

Pathogen whole-genome sequencing (WGS) data, coupled with epidemiological data from hosts, have been used to infer patterns of infectious disease transmission in communities. The quality of transmission inference is linked to the completeness of sampling. For many infectious diseases, there are practical challenges that limit the completeness of such datasets. Recent work demonstrated that a regression-based approach to identify transmission cluster members, when WGS data are missing for a subset of tuberculosis (TB) cases, performed well in a relatively low burden setting in Spain. In this article, we apply this approach to data from Moldova where TB is more widespread. We find that such methods could not accurately identify transmission cluster members, suggesting the need for more robust methods in high TB incidence settings.

## Data Summary

All sequencing data in this study are available in BioProject (https://www.ncbi.nlm.nih.gov/bioproject/PRJNA736718). Individual accession numbers for sequences used in this analysis (*n* = 1,582) are included in a supplementary file (Table S1, available in the online Supplementary Material). We use the R package ‘lr2cluster’ to produce the results reported in this study. All scripts and data that outline our findings can be found on Github (https://github.com/i-rancu/PLRModel_manuscript).

## Introduction

The application of whole-genome sequencing (WGS) has allowed for high-resolution typing and drug-resistant profiling of *Mycobacterium tuberculosis *sequences, transforming our understanding of the transmission [1] and global dispersion of tuberculosis (TB) [2,3]. An important use of WGS data to infer transmission is by grouping similar sequences into clusters, which may be indicative of recent transmission between hosts [4]. The simplest way to assign individuals with TB to clusters is based on the number of single nucleotide polymorphisms (SNPs) by which two strains differ [1,5]. Once clusters are defined, demographic and epidemiological data can be leveraged to understand factors associated with belonging to the same cluster [1,6] and to support public health interventions [7].

One challenge of most molecular epidemiological approaches is the need to isolate mycobacterial DNA from culture prior to sequencing. Globally, only about 60% of pulmonary *M. tuberculosis* cases are diagnosed based on a positive microbiological test [8]. The high proportion of unsequenced TB infections with microbiological confirmation limits the completeness of WGS datasets, which may compromise inference about transmission in areas where many cases are not culture-positive or for whom sequencing is not performed.

A recent study introduced a pairwise logistic regression (PLR) model for predicting membership in *M. tuberculosis* transmission clusters using data collected in Valencia, Spain [9], a country with low TB incidence in which a large fraction of cases occur among the foreign-born. Here, we adapted this model and applied it to data collected in a large country-wide study of TB transmission in the Republic of Moldova. The objective of this analysis was to determine whether a PLR approach can accurately predict cluster membership in a setting where the majority of TB transmission occurs locally.

## Methods

### Data and approach

We used data from a previously reported country-wide molecular epidemiology study conducted in the Republic of Moldova in 2018–2019 [10]. We recruited all notified patients with TB and performed WGS on all culture-positive diagnostic specimens from consenting participants, along with collecting associated host demographic information (Table 1) [10]. We mapped sequencing reads to the H37Rv reference strain (Accession NC_000962.3) using *BWA* and performed variant calling with *GATK* to identify SNPs. A final multi-sequence alignment of concatenated SNPs (41,160 sites) was used to construct a maximum-likelihood phylogeny with *IQ-Tree* after excluding SNPs in repetitive regions, PE/PPE genes, and known resistance-conferring genes [11]. The R package *TreeCluster* [12] was used to identify putative transmission clusters using three patristic distance thresholds, which represent the maximum phylogenetic distance between cases in the same cluster: 5e-4, 1e-3 and 2e-3 substitutions/sites corresponding to ~20, ~40 and ~80 SNPs, respectively.

**Table 1.** Table of epidemiological data of unclustered and clustered individuals with an SNP threshold of ~40 with no missingness in predictor variables

		All individuals*~40 SNPs*(*N=1582*)		Clustered individuals*~40 SNPs, size k=3*(*N=1183*, 74.8%)	Unclustered individuals*~40 SNPs, size k=3*(*N=399,* 25.2%)	Clustered individuals*~40 SNPs, size k=10*(*N=808*, 51.1%)	Unclusteredindividuals*~40 SNPs, size k=10*(*N=729*, 48.9%)
**Gender**							
	Female	365 (23.1%)		270 (22.8%)	95 (23.8%)	195 (24.1%)	162 (22.2%)
	Male	1,217 (76.9%)		913 (77.2 %)	304 (76.2 %)	613 (75.9 %)	567 (77.8 %)
**Age**							
	Mean (SD)	43.6 (13.6)		42.8 (13.3)	45.9 (14.3)	42.2 (13.0)	45.2 (14.0)
	Median [Min, Max]	43.0 [0, 86.0]		42.0 [0, 86.0]	45.0 [2.0, 86.0]	41.0 [1.00, 86.0]	44.0 [0, 86.0]
**Previously Incarcerated**							
	No	1,404 (88.7 %)		1,036 (87.6%)	368 (92.2%)	708 (87.6%)	659 (90.4%)
	Yes	178 (11.3%)		147 (12.4%)	31 (7.8%)	100 (12.4%)	70 (9.6%)
**Homeless**							
	No	1,415 (89.4 %)		1,054 (89.1 %)	361 (90.5 %)	732 (90.6 %)	644 (88.3 %)
	Yes	167 (10.6 %)		129 (10.9 %)	38(9.5 %)	76(9.4 %)	85(11.7 %)
**Transnistria**							
	No	1,396 (88.2 %)		1,018 (86.1 %)	378 (94.7 %)	668 (82.7 %)	687 (94.2 %)
	Yes	186 (11.8 %)		165 (13.9 %)	21 (5.3 %)	140 (17.3 %)	42 (5.8 %)
**Urban**							
	No	951 (60.1 %)		686 (58.0 %)	265 (66.4 %)	448 (55.4 %)	474 (65.0 %)
	Yes	631 (39.9 %)		497 (42 %)	134 (33.6 %)	360 (44.6 %)	255 (35.0 %)

Proportion of individuals within each categorical predictor and the distribution within continuous predictors are reported along with the number of clustered individuals that exist at the k=3 and k=10 size cutoffs*.*

In total, 2,220 (79 %) *M*. *tuberculosis* strains were successfully sequenced; 386 had evidence of polyclonal infection and were excluded from the analysis and the remaining 1834 were used to infer clusters. A total of 1,548 (84%) of these isolates were in putative transmission clusters using a relatively permissive cluster definition (maximum pairwise distance of 1e-3 substitutions/site or ~40 SNPs), ranging in size from 2 to 105 isolates (Fig. S1). For subsequent analysis, we restricted our dataset to all cases, regardless of cluster membership, that had no missing information for all predictor variables used in our models (*n*=1,582; Table 1).

### Pairwise logistic regression

We investigated whether we could predict which (if any) transmission cluster an *M. tuberculosis* strain would be assigned based on available epidemiological, demographic and spatial data. We predicted the transmission cluster to which a case was most likely to belong to using a relatively permissive cluster definition (~40 SNPs maximum) and two minimum cluster sizes: 3 and 10 individuals. For each minimum cluster size *k*, any individual in a cluster size *n*<*k* (including individuals with no putative cluster) was labelled ‘unclustered’. We then partitioned the data into 10 unique test sets (each comprising of 10 % of the data) and 10 training sets (each comprising the complementary 90% of the data), ensuring the test set never contained every member of a transmission cluster.

We fit a PLR model to all unique pairs in the training set with the outcome of 1 if that pair of individuals belong to the same cluster and 0 if not. In addition to the predictor variables described in Table 1, we used the distance between the individuals’ *reguine* (lowest administrative unit) of residence. We used the resulting parameter estimates to predict cluster membership for all cases in the test dataset. We report predictions for both the single most probable cluster (‘best 1’) and the three most probable clusters (‘best 3’) for each case. The model was considered ‘correct’ if the individual’s true cluster is the ‘best 1’ or one of the clusters in the ‘best 3’. We repeated this analysis ten times with each unique test set. Model accuracy was computed as the fraction of new cases whose true cluster was predicted correctly [9], and we expect that individuals labelled ‘unclustered’ will never be correctly assigned to a genomic cluster. We then compared the PLR model accuracy to an approach where we randomly assigned isolates to transmission clusters with probabilities proportional to cluster sizes.

### Sensitivity analyses

We repeated the PLR model but only included individuals in clusters of size *n*≥k (i.e., we excluded any ‘unclustered’ individuals from the training and test sets). In addition, to test the effect of the genomic cluster threshold on model accuracy, we repeated the analysis using more permissive (maximum pairwise distance of 2e-3 substitutions/site or ~80 SNPs) and restrictive (5e-4 substitutions/site or ~20 SNPs) thresholds for clustering.

All analyses were conducted using R software, version 4.3.2.

## Results

We implemented the PLR model using training and test sets derived from all individuals, considering clusters with a maximum pairwise distance of ~40 SNPs and a minimum cluster size of k=3. The most influential predictors were if both individuals in the pair were in Transnistria (standardized regression parameters: 1.11), if they were previously incarcerated (0.56) and a smaller spatial distance between individuals (−0.24). We found that the model predicted the true cluster membership of cases in the test set with a mean accuracy of 4.24% for the ‘best 1’ approach and 10.7% for the ‘best 3’ approach. With a k=10 threshold, we found that model performance worsened, yielding a mean accuracy of 3.10% for ‘best 1’ approach and 8.98% for ‘best 3’ approach. Despite low accuracy, the PLR model outperformed random assignment, which is expected to have low accuracy as it assigns individuals to a putative transmission cluster with a probability proportional to cluster size.

**Fig. 1.**
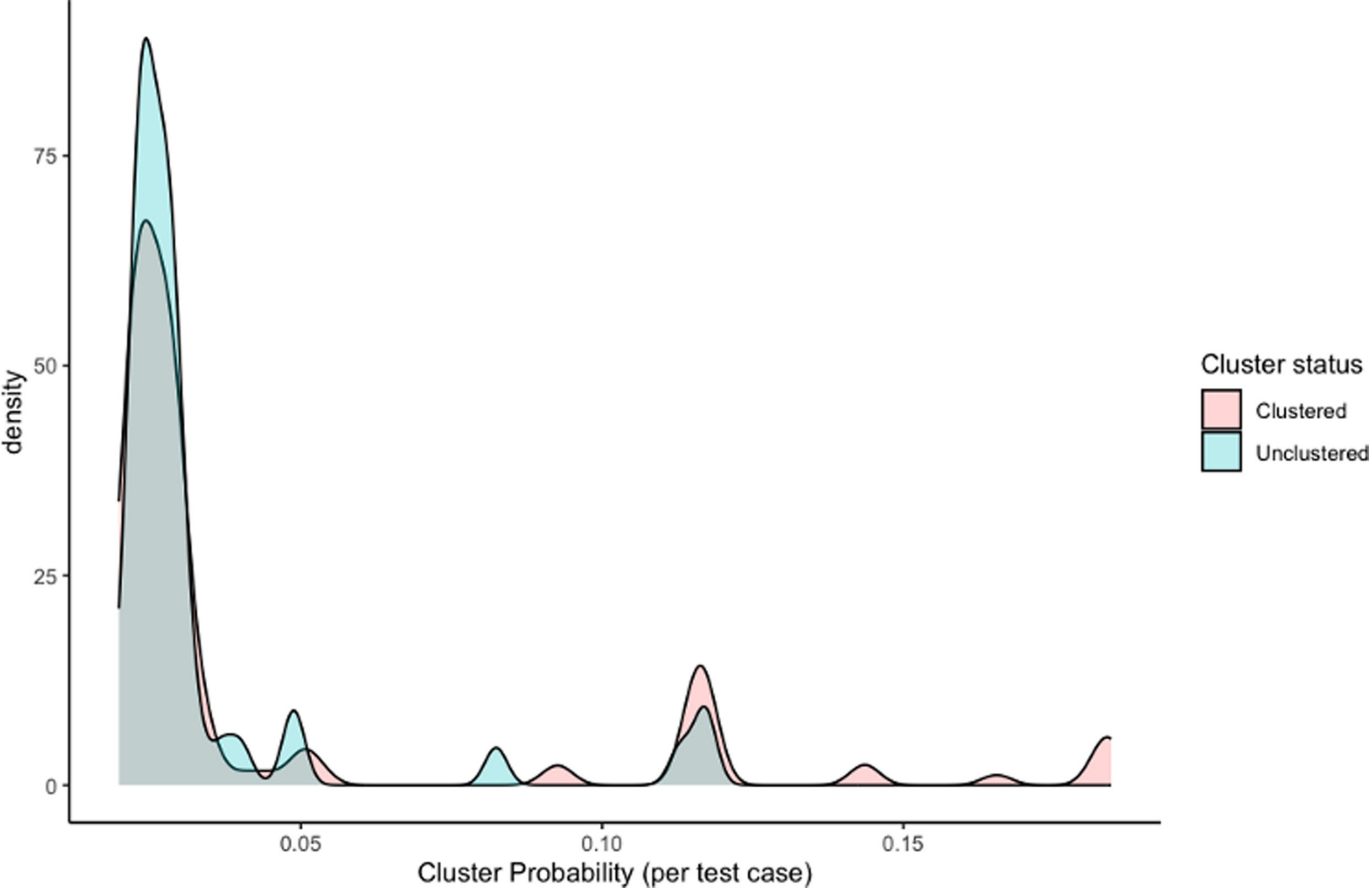
Example probability distribution associated with ‘best 1’ predicted clusters for all individuals in a sample test set for cluster size definition k=3 and ~40 SNPs threshold. Probabilities are coloured by whether the individual in the test set was labelled as a clustered (belonging to a genomic cluster size n≥3) or unclustered (belonging to a genomic cluster size n≤2 or no cluster at all).

In this simple implementation, a truly unclustered individual could never have a ‘correct’ cluster assignment, as they were always assigned to a cluster based on their highest pairwise probability. An alternative approach would be to identify a probability threshold below which an individual would not be assigned to a cluster, which can allow for unclustered individuals to be correctly identified. However, we found that the probabilities associated with the ‘best 1’ cluster for truly unclustered and truly clustered individuals in the test set overlapped substantially, suggesting that clustered individuals were not substantially more likely to be assigned to a cluster than unclustered individuals (Fig. 1).

**Fig. 2.**
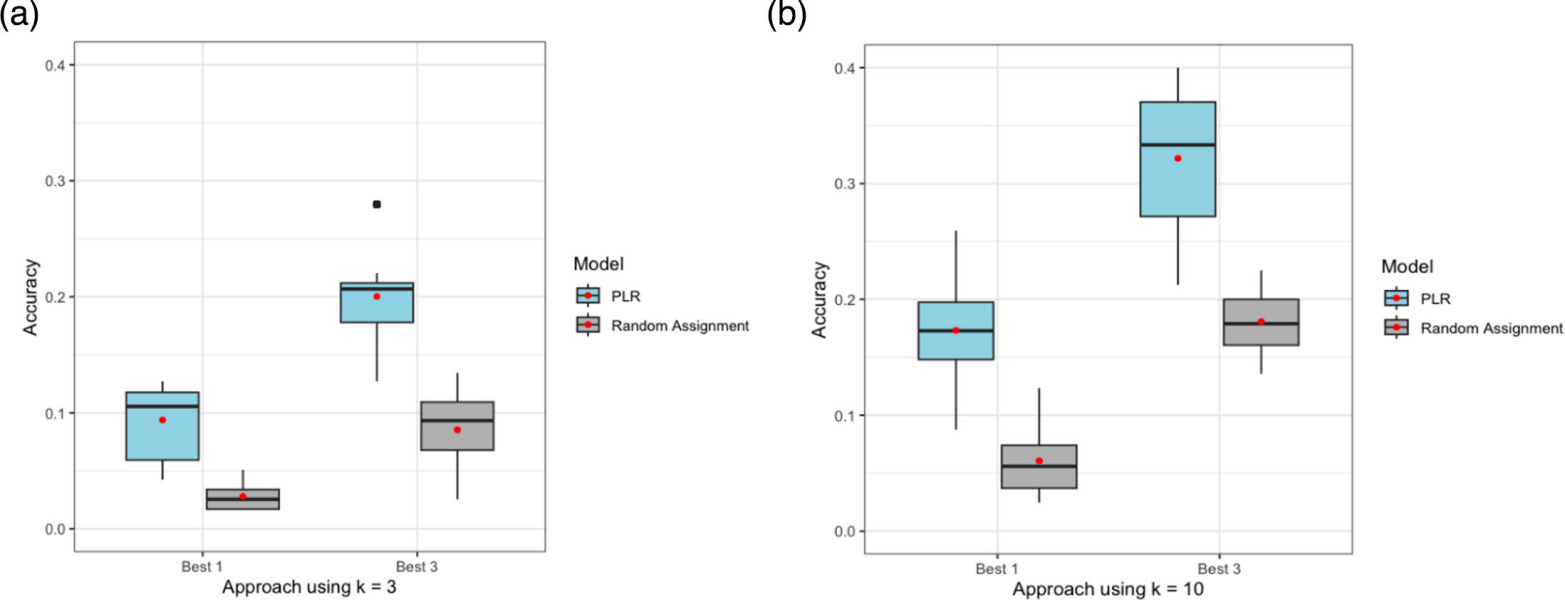
PLR model accuracies for ‘best 1’ and ‘best 3’ approaches using a threshold of ~40 SNPs and cluster size threshold of (**a**) k=3 and (**b**) k=10. PLR model performance was compared to random assignment, which assigns a likely genomic cluster with a probability proportional to cluster size. Model accuracies are computed as the average over the 10 unique test sets, with the mean accuracy indicated relative to the median by a red dot.

We next ran the model including only individuals in clusters determined by a maximum threshold of ~40 SNPs. For the k=3 threshold (1,183 individuals in 107 clusters), we found that the model predicted the true cluster membership of individuals in the test set with a mean accuracy of 9.55% for the ‘best 1’ approach and 21.8% for the ‘best 3’ approach (Fig. 2a). For the k=10 threshold (808 individuals in 27 clusters), we found that model accuracy almost doubled to 17.2% for ‘best 1’ approach and 31.2% for ‘best 3’ approach (Fig. 2b). Both approaches outperformed random assignment (Fig. 2).

To determine whether these results were dependent on cluster definition, we refit the model using clusters found using maximum thresholds of ~20 SNPs and ~80 SNPs (Table 2). For the ~20 SNP threshold, we estimated an accuracy of 8.97% for ‘best 1’ approach and 18.8% for ‘best 3’ approach at the k=3 cutoff (Table 2). The model performed similarly for the ~80 SNP threshold, yielding 8.22% for the ‘best 1’ and 19.2% for the ‘best 3’ approach (Table 2). Increasing the minimum cluster size to k=10 increased model accuracy, yielding 17.0% and 12.6% for ‘best 1’ using ~20 and ~80 SNPs, respectively (Table 2). All sensitivity analyses using the PLR model outperformed random assignment (Table 2).

**Table 2.** Table of accuracies for all genomic cluster thresholds and approaches with accuracy reported for both PLR model and random assignment

Cluster size cutoff	SNP threshold	PLR, ‘best 1’ Accuracy (%)	PLR, ‘best 3’ Accuracy (%)	Random,‘best 1’Accuracy (%)	Random,‘best 3’Accuracy (%)	No. clusters	No.ind.
k≥3	20	8.97	18.8	1.49	2.98	136	804
	40	9.55	21.8	3.05	8.55	107	1183
	80	8.22	19.2	3.50	12.1	95	1266
k≥10	20	17.0	39.1	7.82	22.0	18	282
	40	17.2	31.2	5.81	20.2	27	808
	80	12.6	28.8	6.61	19.6	26	938

The number of genomic clusters and individuals included in each respective analysis are reported for each sensitivity analysis*.*

## Discussion

We adapted and applied a pairwise regression approach to predict which putative transmission cluster an individual with TB most likely belongs to. When training the PLR model on both unclustered and clustered individuals, the method failed to distinguish the two types of individuals, resulting in very low model accuracy. When restricting analyses to only clustered individuals, model accuracy improved marginally. We found that the accuracy of the PLR approach was not sensitive to the choice of maximum pairwise distance thresholds but it was sensitive to the choice of minimum cluster size. Model accuracy improved when we used a cluster size cutoff of k=10, and we hypothesize that this improvement comes from reducing the total number of clusters (Fig. S1).

Notably, restricting the analysis to large clusters did not increase accuracy to levels described in the previous application of this approach to *M. tuberculosis in* Valencia [9]. In Valencia, the majority of incident TB occurs in high-risk populations, and including data on nationality, HIV status and diabetes diagnosis improved cluster prediction. While we did not have these data from Moldova, their exclusion likely had a modest impact on model performance in an endemic setting where TB transmission is not limited to high-risk populations. Additionally, the Valencia dataset included participant’s home addresses, while the data from Moldova included home village or city borough, limiting the precision of distance between cluster members as a predictor in our model.

A limitation of this application is the poor classification of individuals who do not belong to any putative transmission cluster. Future analyses may consider a multinomial approach in which pairs are classified into one of three categories: both clustered, both unclustered or mixed pairs. This approach would not enable the identification of the specific cluster to which a case most likely belongs; rather, it would need to be the first in a two-stage approach to classify unsequenced pairs.

Moldova is a TB-endemic setting and transmission is widespread throughout the country [13]. While we found that there were weak associations between specific genomic clusters and factors such as case home location, incarceration history and homelessness, transmission cluster membership was not easily predicted from routinely collected data. Our analyses suggest that while the PLR approach may perform well in lower burden settings where most transmission occurs within distinct subpopulations, we find that this approach does not allow for accurate cluster membership prediction in Moldova, where multiple strain types are co-circulating [13].

## Conclusion

In the present study, we demonstrate that a pair-wise logistic regression approach to classify unsequenced strains in a region of endemic TB does not yield high-accuracy cluster assignments. We found that changing the definition of a cluster (i.e. changing the SNP threshold for cluster membership) had a minimal effect on model accuracy, while increasing the minimum cluster size (i.e. restricting the data to larger clusters only) increased model accuracy. These findings highlight the challenges of classifying individuals into clusters in endemic TB regions.

## Supplementary material

10.1099/acmi.0.000964.v3 Fig. S1.

10.1099/acmi.0.000964.v3 Table S1.

## Abbreviations

*M. tuberculosis*: *Mycobacterium tuberculosis*
PLR: pairwise logistic regression
SNP: single nucleotide polymorphism
TB: tuberculosis
WGS: whole-genome sequencing
