# Supplementary material for: Classification of unsequenced Mycobacterium tuberculosis strains in a high-burden setting using a pairwise logistic regression approach
Source: Access Microbiol. 2025 May 12;7(5):000964.v3. doi: 10.1099/acmi.0.000964.v3 (PMC12163731; doi:10.1099/acmi.0.000964.v3)

Supplementary Figure 1: Distribution of cluster size frequency for all clustered individuals by SNP threshold. Red line demarcations show the n ≥ 3 and n ≥ 10 cluster size cutoffs for the (a) ~80 SNPs, (b) ~40 SNPs, and (c) ~20 SNPs genomic cluster thresholds used in model analyses.

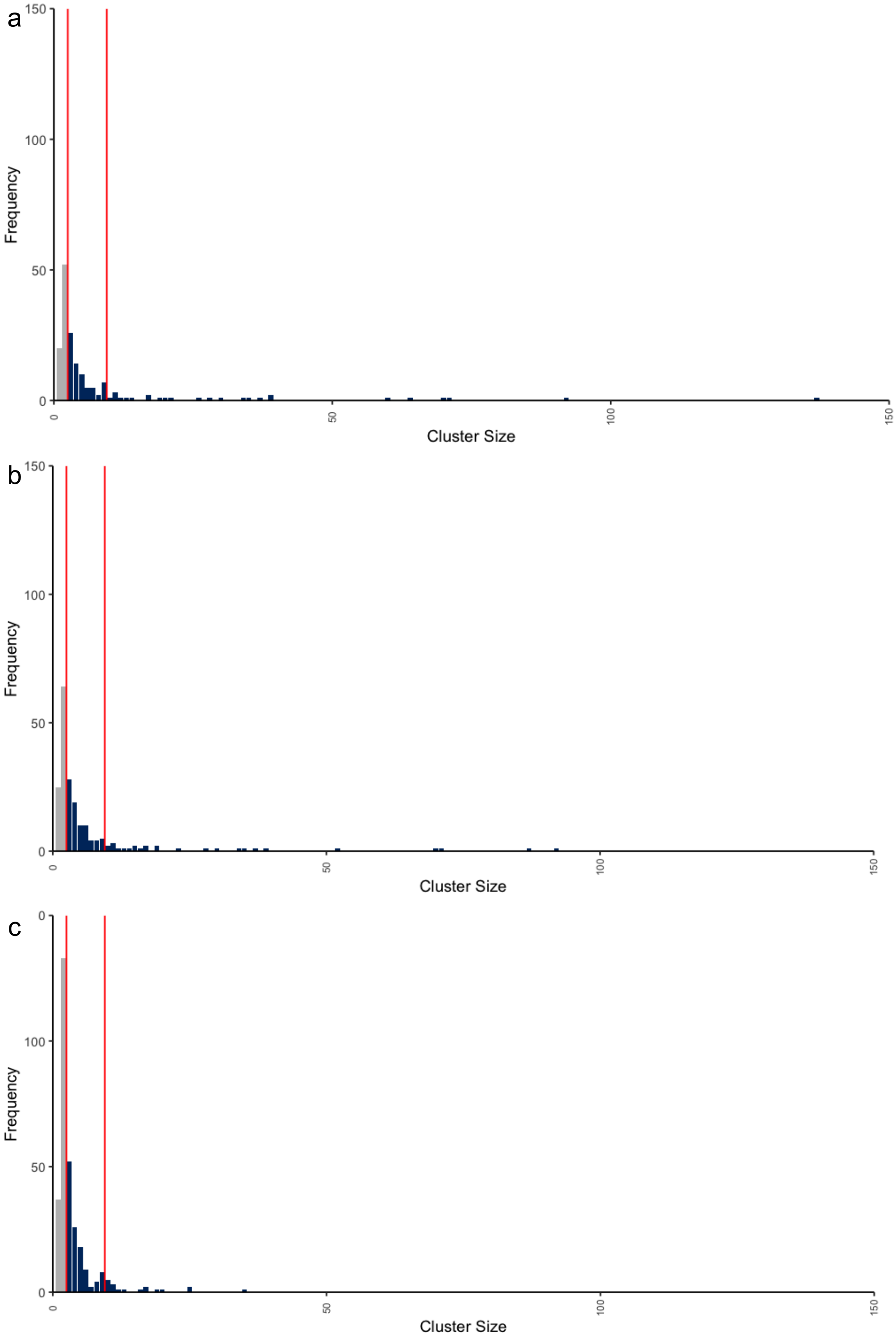

Supplement: Fig. S1. [file acmi-7-00964-s001.pdf]
